# The Mode of Infection in Kala-Azar

**Published:** 1909-01-30

**Authors:** 


### TROPICAL DISEASES.

### THE MODE OF INFECTION IN KALA-AZAR.

FOLLOWING the differentiation from malaria of that disease now called by the native name of kala-azar, the discovery of the peculiar immature Leishman-Donovan trypanosomes in the spleen-pulp has led to vigorous attempts to discover how the parasites are introduced into the human body. Captain W. S. Patton, I.M.S., has lately written a second report upon the subject for the Government of India. In a previous report he had advanced the hypothesis that the parasite is probably transmitted by some blood-sucking insect. There was no evidence to show that this is the mosquito, as in the case of malaria; and Captain Patton's attention was attracted to the Indian bed-bug, *Cimex rotundatus*, within which he found parasites developing. This hypothesis is confirmed in the second report.

The Leishman-Donovan parasites are confined to the spleen only in patients who are neither acutely nor severely afflicted by the disease. In cases of acute kala-azar, on the other hand, and in chronic cases that have reached the last stages, the parasites are to be detected in the peripheral blood, as well as in the spleen, and it is from such cases as these that the bed-bugs become infected, and thus transmit the parasite to the next person they bite. Natives being far less cleanly than Europeans, they suffer the more commonly, but whites are by no means immune.

Captain Patton fed bed-bugs on patients in whom the parasites had been demonstrated in the peripheral blood, and then studied the development of the parasite ingested in the mid-gut of this bug, both sexes of which are blood-suckers. The parasites undergo complete development to the flagellate forms—trypanosomes—in three days, although they may remain dormant in the mid-gut for several days before beginning to develop. Rapid multiplication by rosette formation occurs within the bug.

The pediculus capitis has been observed to contain Leishman-Donovan bodies after sucking blood from a kala-azar patient in a stage at which the parasites were in the peripheral blood; but no development of the ingested parasites occurs in this louse.

